# Exosomes Cause Preterm Birth in Mice: Evidence for Paracrine Signaling in Pregnancy

**DOI:** 10.1038/s41598-018-37002-x

**Published:** 2019-01-24

**Authors:** Samantha Sheller-Miller, Jayshil Trivedi, Steven M. Yellon, Ramkumar Menon

**Affiliations:** 1 Division of Maternal-Fetal Medicine and Perinatal Research, Department of Obstetrics and Gynecology, The University of Texas Medical Branch, Galveston, Texas USA; 2 Department of Biochemistry and Molecular Biology, The University of Texas Medical Branch, Galveston, Texas USA; 3 Longo Center for Perinatal Biology, Departments of Basic Sciences and Pediatrics, Loma Linda University School of Medicine, Loma Linda, CA USA

## Abstract

Endocrine factors and signals of fetal organ maturation are reported determinants of birth timing. To test the hypothesis that paracrine signaling by exosomes are key regulators of parturition, maternal plasma exosomes from CD-1 mice were isolated and characterized throughout gestation and the biological pathways associated with differentially-expressed cargo proteins were determined. Results indicate that the shape and size of exosomes remained constant throughout gestation; however, a progressive increase in the quantity of exosomes carrying inflammatory mediators was observed from gestation day (E)5 to E19. In addition, the effects of late-gestation (E18) plasma exosomes derived from feto-maternal uterine tissues on parturition was determined. Intraperitoneal injection of E18 exosomes into E15 mice localized in maternal reproductive tract tissues and in intrauterine fetal compartments. Compared to controls that delivered at term, preterm birth occurred in exosome-treated mice on E18 and was preceded by increased inflammatory mediators on E17 in the cervix, uterus, and fetal membranes but not in the placenta. This effect was not observed in mice injected with early-gestation (E9) exosomes. This study provides evidence that exosomes function as paracrine mediators of labor and delivery.

## Introduction

Parturition is an inflammatory process involving both fetal and maternal tissues and is initiated by fetal endocrine signals as well as signals arising from organ maturation at term (i.e., around 37–40 weeks of gestation)^1,2^. In humans, the inflammatory signals of fetal readiness for delivery lead to functional progesterone withdrawal^3,4^, the recruitment and activation of immune cells, and the development of an inflammatory overload in the uterine cavity^5,6^, which disrupts the homeostatic factors that maintain pregnancy and leads to the promotion of fetal delivery. Although fetal endocrine signals are a component of the biological clock that signals organ maturation and determines the timing of birth^7–9^, paracrine signaling by intercellular signaling vesicles (called exosomes) may also contribute to the initiation of labor. However, knowledge gaps exist in understanding the signature of paracrine mediators, how they are generated, and how they are propagated to initiate labor and delivery^10,11^. How paracrine mediators regulate cervical remodeling and maturation of uterine contractile capabilities is essential for understanding the premature activation of such factors that are often postulated to be associated with spontaneous preterm birth, which complicates approximately 10.5% of all pregnancies^12–14^.

At term, inflammatory mediators, often referred to as sterile inflammation, that are capable of contributing to labor-associated changes are elevated in both fetal and maternal gestational tissues^15,16^. Senescent fetal (amniochorionic membranes) or maternal (decidua) tissues produce inflammatory markers^17–20^ termed the senescence-associated secretory phenotype (SASP)^21,22^ as part of the molecular mechanism for sterile inflammation^23–25^. In addition to SASP, senescent fetal cells release damage-associated molecular patterns (DAMPs)^24,26^. SASP and DAMPs are postulated to constitute a set of sterile inflammatory signals that can be propagated from fetal to maternal tissues to indicate fetal readiness for delivery^27^. In addition, this inflammatory overload in maternal gestational tissues can create labor-associated changes^16,28,29^. Unlike endocrine mediators, senescence and the senescence-associated development of inflammatory paracrine signaling are similar in both human and rodent pregnancy and labor, thus suggesting that natural and physiological fetal tissue aging is an independent process and is unlikely to be regulated by endocrine mediators of pregnancy^30–32^. Senescence of the fetal membrane tissues is a physiological event in fetal membranes throughout gestation and is well correlated with fetal growth and organ maturation. Oxidative stress that builds up in the amniotic cavity at term accelerates senescence and the production of senescence-associated sterile inflammation^33,34^ and this mechanism is considered as a contributor to labor and delivery.

The propagation of sterile inflammatory signals between fetal and maternal tissues can occur as simple diffusion through tissue layers or, more efficiently and in a protected manner, through extracellular vesicles (e.g. exosomes)^35^. Exosomes are 30–150 nm membrane vesicles that are formed by the inward budding of the late endosome^36,37^. Exosomes are released by cells and carry cellular metabolic byproducts including, but not limited to, proteins, nucleic acids, and lipids, and they represent the metabolic state of the cell that releases them^38,39^. Thus exosomes represent the biological and functional state of the origin cell, and studying them can provide evidence for the underlying status of the organ^40,41^. Evidence suggests that exosomes play a role in the paracrine communication between fetal and maternal tissues. Specifically, (1) senescent fetal cells produce exosomes and carry fetal specific markers, SASPs, and DAMPs^38,42^; (2) irrespective of the experimental conditions (normal cell culture vs. oxidative stress conditions), exosomes carry inflammatory mediators; however, the inflammatory markers are unique depending on the type of treatment^43^; (3) fetal-derived exosomes can traffic from the fetal to the maternal side^35^; and (4) fetal exosomes may be capable of causing inflammatory activation in maternal gestation cells (myometrium and decidua) but not in placental cells. Besides this data, the current literature on exosomes during pregnancy has focused on placental exosomes and their potential effect on maternal tissues^31,44–47^. Several studies have explored the biomarker potential of exosomes and their cargo in adverse pregnancies, including preeclampsia and diabetes^48–52^. Although these descriptive observations suggest a courier or biomarker role for exosomes, a functional role of exosomes was lacking in processes associated with parturition or pregnancy-associated pathologies. Therefore, the primary objective of this study was to test the hypothesis that late gestational exosomes induce preterm parturition in mouse models of pregnancy. Little is known about exosomes during normal mouse pregnancy, therefore total maternal plasma exosomes were also characterized at various stages of mouse gestation prior to testing the functional role.

## Results

### Plasma exosomes exhibit classic exosome characteristics

**Figure 1:**
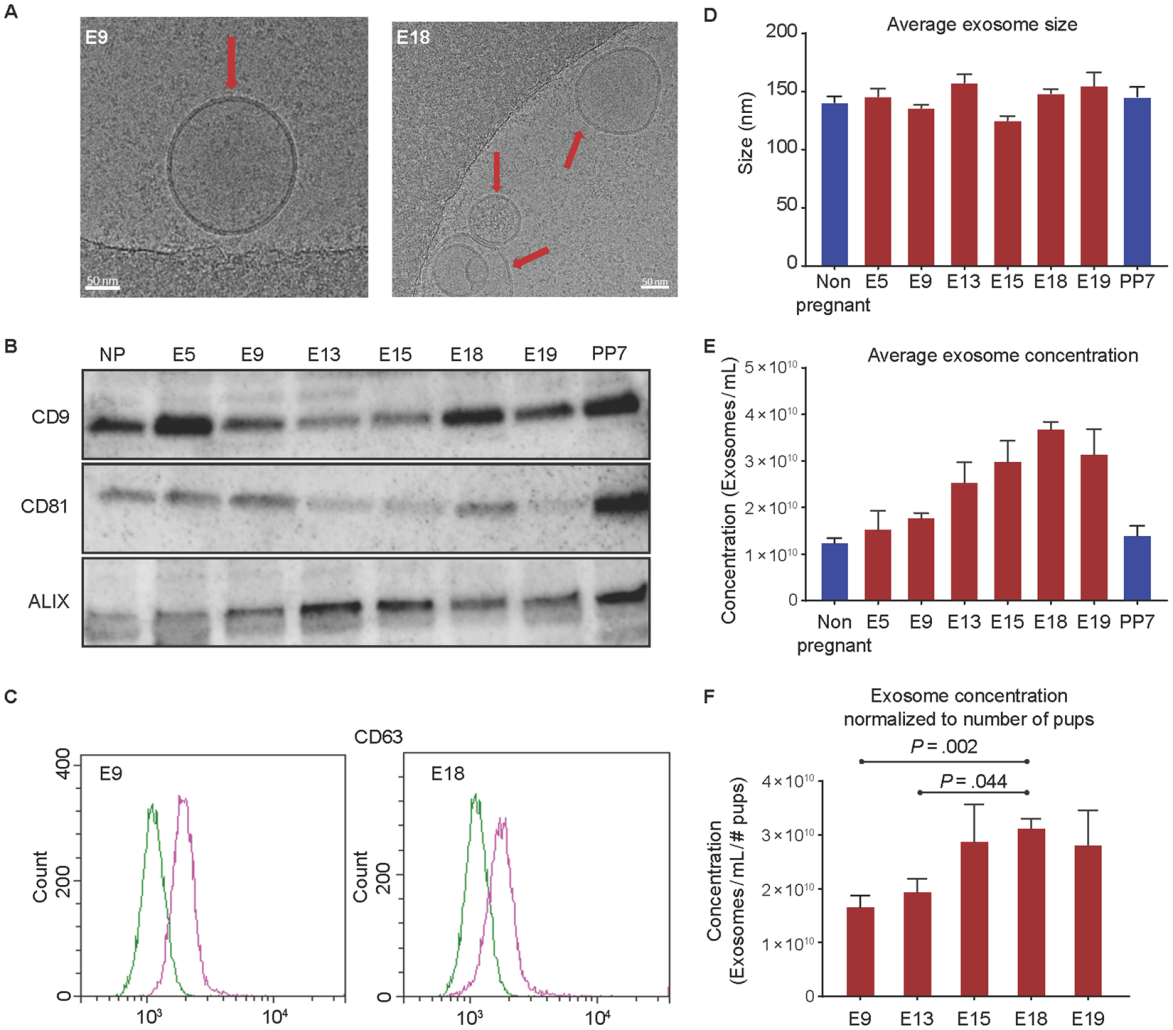
Characterization of exosomes isolated from maternal plasma. (**A**) A representative cryo-electron microscopy image showing classic exosome characteristics of double-membrane vesicles from early gestation (E9) and late gestation (E18). The arrow indicates exosomes, and the scale bar represents 50 nm. (**B**) Exosomes from maternal plasma were lysed and analyzed for exosome markers using western blot analysis. Regardless of gestation day or pregnancy status, there was consistent expression of exosome markers CD9, CD81, and ALIX. Full-length blots are presented in Supplemental Fig. 2. (**C**) Representative flow cytometry histograms for maternal plasma exosome marker CD63 from E9 and E18 exosomes. X-axis, FITC intensity; y-axis, count or the number of beads positive for exosomes. Green represents the negative control; pink represents beads containing exosomes positive for CD63. (**D**) Exosome size was determined using nanoparticle tracking analysis and was not significantly different between gestation days. (**E**) Nanoparticle tracking analysis was performed to determine concentration of plasma exosomes throughout gestation. The average exosome concentration increased significantly throughout gestation (E5 through E19) compared to the NP state. The maximum number of exosomes were seen on day 18. (**F**) The average exosome concentration for each gestation day was normalized to the average number of pups per mouse to determine if the increase in exosome concentration throughout gestation was dependent on the number of pups each mouse was carrying. Exosome concentration per pup on E18 was still the maximum concentration observed. Exosome concentration per pup was significantly higher on E18 compared to E9 and E13 when adjusted for the number of pups.

To understand the changes associated with exosome quantity and characteristics, total exosomes were isolated from maternal plasma samples at various gestational days and compared to nonpregnant (NP) and postpartum day 7 animals. Regardless of pregnancy or gestation day, exosomes isolated from maternal plasma were round double-membrane vesicles (Fig. 1A). Western blot analysis indicated that exosomes contained tetraspanin markers CD9 and CD81, as well as the multivesicular body protein ALIX (Fig. 1B). As evidenced by flow cytometry, exosomes were also positive for another tetraspanin exosome marker, CD63 (Fig. 1C).

### Exosome concentration increases throughout gestation

Exosomes isolated from maternal plasma did not differ significantly in size throughout gestation (Fig. 1D). However, a significant increase in exosome quantity was seen between E5 (1.52 × 10^11^ exosomes/mL) and E19 (3.13 × 10^11^ exosomes/mL), with the maximum concentration seen at E18 (3.66 × 10^11^ exosomes/mL, Supplemental Table 1 and 2) (Fig. 1E). The trend remained the same after normalizing to the number of pups, with E18 showing the maximum number of exosomes (Supplemental Table 1). As shown in Fig. 1, significant differences were noted (Fig. 1F) between E18 and E9 (*P* = 0.002) and E18 and E13 (*P* = 0.044).

### Proteomic analysis of maternal plasma exosomes throughout gestation

**Figure 2:**
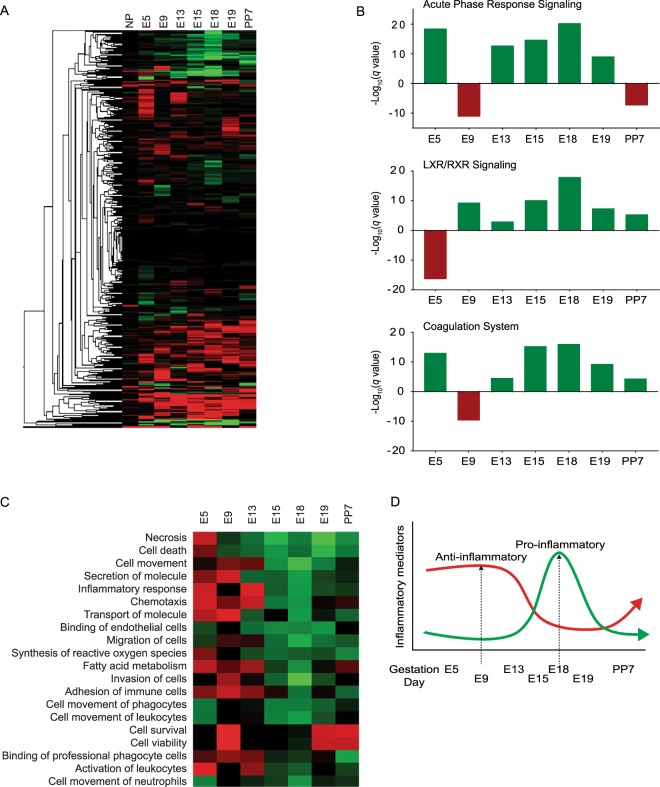
Proteomic and bioinformatic analysis of maternal plasma exosomes throughout gestation. All fold change values were relative to NP exosomes. For IPA, proteins included had ±1.5-fold change relative to the NP controls and a *P*-value < 0.01. (**A**) Heatmap to visually represent differentially-expressed exosome proteins (912) throughout gestation. Green is an increased fold change, while red is a decreased fold change. (**B**) Graphical representation of the top three canonical pathways identified during late gestation: acute-phase response signaling (top), liver X receptor/retinoid X receptor (LXR/RXR, middle), and coagulation (bottom). These pathways are associated with inflammation and increased between E13–E18, peaked on E18, then decreased postpartum. The x-axis represents gestation day, and the y-axis represents −log (q-value). Green represents a positive Z-score (upregulation), while red represents a negative Z-score (downregulation). (**C**) Heatmap of the top biological functions associated with exosomal cargo on each gestation day shows inflammatory pathways and recruitment of leukocytes and other immune cells increasing throughout gestation, peaking on E18, and decreasing postpartum. Green represents a positive Z-score (upregulation), while red represents a negative Z-score (downregulation). (**D**) Graphical representation of well-reported inflammatory changes during mouse pregnancy, which are also reflected in exosomes throughout gestation. Gestation days are not drawn to scale.

Proteomic analysis of maternal plasma exosomes identified 1283 differentially-expressed proteins. A heat map (Fig. 2A) was created of the 912 differentially expressed proteins that were used for bioinformatics analysis using IPA. Proteomic analysis of exosomal cargo determined that molecules involved with inflammation were the predominant pathway that increased during late gestation. Specifically, acute-phase response signaling, liver X receptor/retinoid X receptor (LXR/RXR), and coagulation canonical pathways (Fig. 2B) increased between E13 and E18, peaked at E18, and returned to levels seen in nonpregnant animals in the postpartum samples (Supplemental Table 3). Analysis of the top biological functions associated with exosomal cargo on each gestation day (Fig. 2C) included inflammatory pathways related to increased recruitment of leukocytes and other immune cells throughout gestation, peaking on E18 and decreasing postpartum. The biological functions associated with the proteins identified in late-gestation exosomes included chemotaxis, the inflammatory response, cell movement, leucocyte activation, and neutrophil infiltration, all of which have been linked to physiological changes associated with prepartum processes related to cervix remodeling and initiation of labor^53–56^ (Fig. 2D).

**Figure 3:**
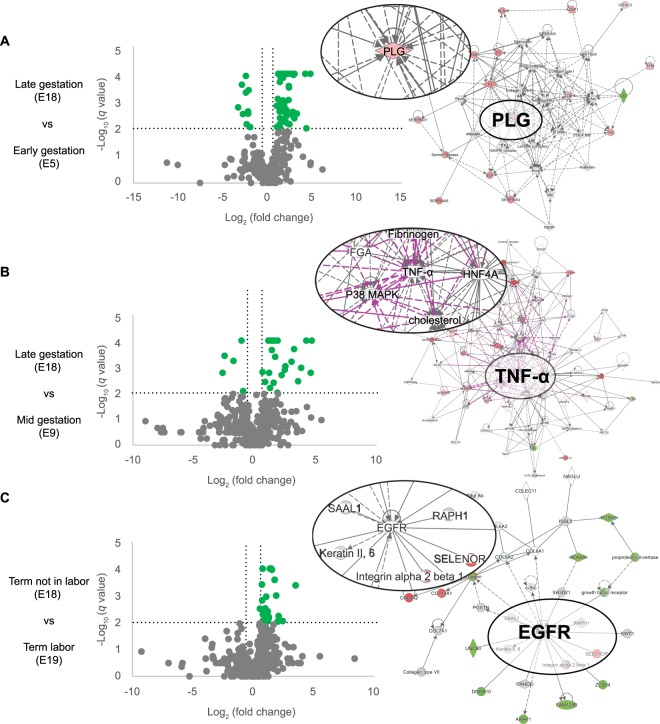
Volcano plots and molecular networks of differentially-expressed proteins. Molecular networks were identified using Ingenuity Pathway Analysis (IPA) based on proteins with a ±1.5-fold change (log_2_ fold change = ±0.6) and a q-value < 0.01 (−log q-value = 2). Circular insets are a zoomed in view of the central molecules identified. (**A**) Comparison of E18 (late gestation) and E5 (early gestation) identified plasminogen (PLG) as a central molecule in the molecular network. (**B**) Comparison of E18 and E9 identified TNF-α as a central molecule in the molecular network. (**C**) Comparison of E18 (term not in labor) and E19 (term in labor) exosome cargo identified epidermal growth factor receptor (EGFR) as the central molecule.

The specific scenarios associated with different gestation points were evaluated by investigating the molecular networks that were activated by proteins with a ±1.5-fold change (log_2_ fold change = ±0.6) and a q-value < 0.01 (−log q-value = 2). When comparing E18 (late gestation) to E5 (early gestation), plasminogen (PLG) was identified as a central molecule in the molecular network (Fig. 3A). PLG activates matrix-degrading enzymes MMP-2 and MMP-9, as well as TGF-β. These molecules have been implicated in parturition at term and in preterm birth (Hui *et al*.^57^; Keren-Politansky *et al*.^58^). TGF-β is also a major mediator of epithelial-mesenchymal transition, a mechanism that has been implicated in labor^59,60^. Identified proteins are listed in Supplemental Table 3. E18 was also compared to E9 (Fig. 3B) as these two gestation days seemed to have the most differences in canonical pathways and biological processes. TNF-α was identified as a central molecule in the network comparing exosomal proteins on E18 to E9. TNF-α is a pro-inflammatory cytokine shown to be upregulated in the myometrium at term and can activate smooth muscle contractions^28^. Additionally, TNF-α can activate pro-senescence marker p38 MAPK in epithelial cells^43^. Identified proteins are listed in Supplemental Table 4. Exosome cargo were also compared between E18 (term not in labor) and E19 (term in labor) (Fig. 3C) and the central molecule identified was epidermal growth factor receptor, which activates the mitogen-activated protein kinase family (i.e., ERK, JUN, and p38) and other inflammatory pathways (i.e., STAT3 and the NF-κB complex). Multiple data have shown the mechanistic roles of these factors in term labor^2,61–64^. Identified proteins are listed in Supplemental Table 4.

In summary, the exosomal cargo reflected a gradual buildup of inflammation during the later stages of pregnancy and nonspecific inflammation peaking on the penultimate day before the expected delivery in this mouse model. Delivery day exosomal cargo was characterized by senescence, necrosis, and other cell death pathways and markers as expected^34^. All these indicators returned to normal in postpartum animals. These data suggest that exosomal proteins reflect distinct phases of pregnancy and functional changes that are activated to promote labor and delivery.

### Intraperitoneal injections of E18 exosomes induce preterm birth independent of systemic progesterone withdrawal or inflammation

To determine the role of late-gestation exosomes in labor, exosomes from E18 mice were injected into pregnant mice on E15. E18 was chosen based on data that showed that E18 (the penultimate day of labor and delivery in CD-1 mouse models) had the maximum number of exosomes carrying nonspecific inflammatory mediators. Therefore, the hypothesis that an increased quantity of inflammatory cargo may contribute to labor and delivery was tested in this model. Exosomes from E9 mice were chosen as the control group as they contained a low number of exosomes and a minimal number of inflammatory mediators (Figs 1 and 2). In support of the abovementioned hypothesis, 80% (4/5) of mice injected with E18 exosomes delivered preterm (Supplemental Fig. 1B), which was defined as the delivery of at least one pup on or before E18. Preterm labor occurred 48 hours after the final injection of E18 exosomes (E16). Conversely, E9- and PBS-injected mice (Supplemental Fig. 1A) delivered on E19.5 (Table 1). The number of pups was not different between E18-, E9-, or PBS-injected animals (data not shown). All pups were viable upon delivery and had normal weaning periods as they were born close to term. LPS-injected animals served as positive controls in the preterm labor and delivery experiment, as all E15 LPS-injected mice delivered within 6–12 h and had no viable pups upon delivery.

**Table 1:** Preterm birth rates in PBS, E9 and E18 exosome injected mice.

Treatment	Number of Animals	PTB	Term Birth	PTB (%)
PBS	5	0	5	0%
E9 Exosomes	5	0	5	0%
E18 Exosomes	5	4	1	80%

**Figure 4:**
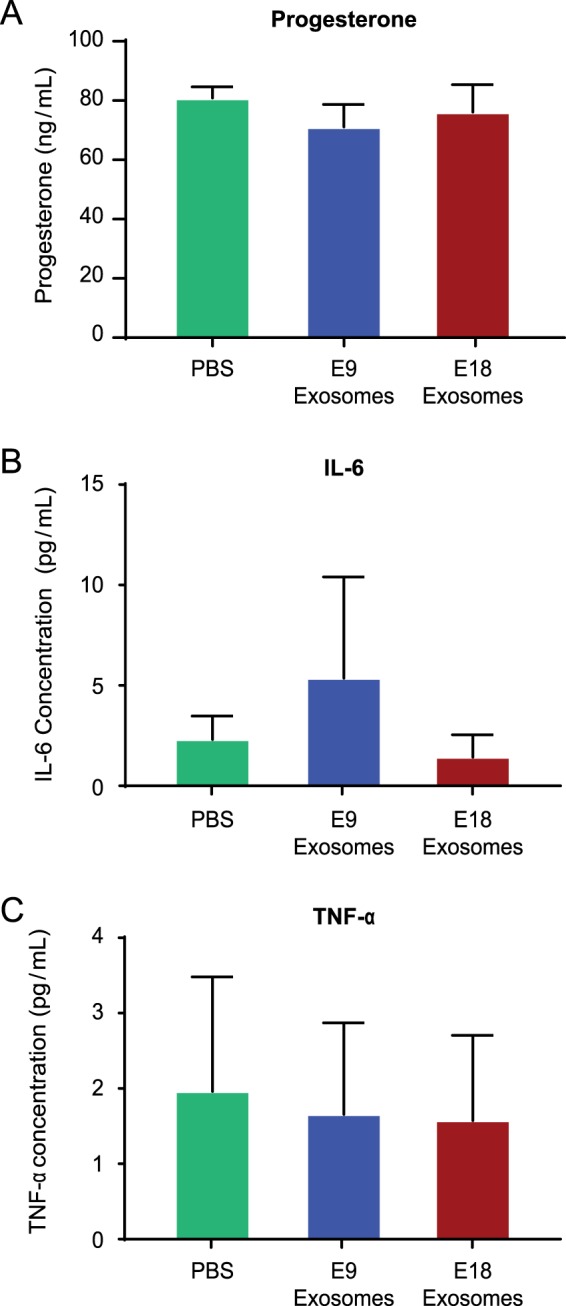
Exosome-induced preterm labor is not associated with systemic progesterone withdrawal or systemic inflammation. (**A**) Plasma progesterone levels were not significantly different regardless of the treatment. (**B**) Bioplex analysis of maternal plasma showed no significant difference in for IL-6 levels in PBS, E9, and E18-injected mice. (**C**) Bioplex analysis of maternal plasma showed no significant difference in TNF-α levels in PBS, E9, and E18-injected mice.

Although systemic progesterone withdrawal is a key mechanistic event in mice that contributes to inflammation^3,65,66^, exosome-mediated preterm labor was not associated with systemic progesterone withdrawal or an increase in key inflammatory markers in circulation. Maternal plasma progesterone levels did not differ between mice injected with PBS and E9 exosomes (76.16 ± 7.09 vs. 70.07 ± 23.88 ng/mL; *P* = 0.83), PBS and E18 exosomes (89.73 ± 5.57 pg/mL; *P* = 0.43), or E9 and E18 exosome (*P* = 0.20) (Fig. 4A). Although E18 exosomes isolated from maternal plasma carry mediators of inflammation, injecting these exosomes did not result in systemic maternal inflammation. As shown in Fig. 4, there were no significant differences in the levels of the inflammatory cytokines, IL-6 and TNF-α (Fig. 4B, all *P* > 0.98 and Fig. 4C, all *P* > 0.65, respectively), in maternal plasma regardless of the treatment. Exosomes not only encapsulate degradation-susceptible signals, but they also carry these signals to specific sites to induce a localized, rather than a systemic, inflammatory response. The tropism exhibited by exosomes was not a part of this study, and the chemotactic signals attracting inflammatory exosomes to specific uterine sites were not investigated. It is speculated that inflammatory mediators required for parturition are protected and delivered at sites where they have specific functions to avoid systemic inflammation that can be detrimental to the maintenance of pregnancy.

### Exosome trafficking to intrauterine tissues to cause labor-associated changes

#### CFSE-labeled exosomes localize in maternal and fetal tissues

**Figure 5:**
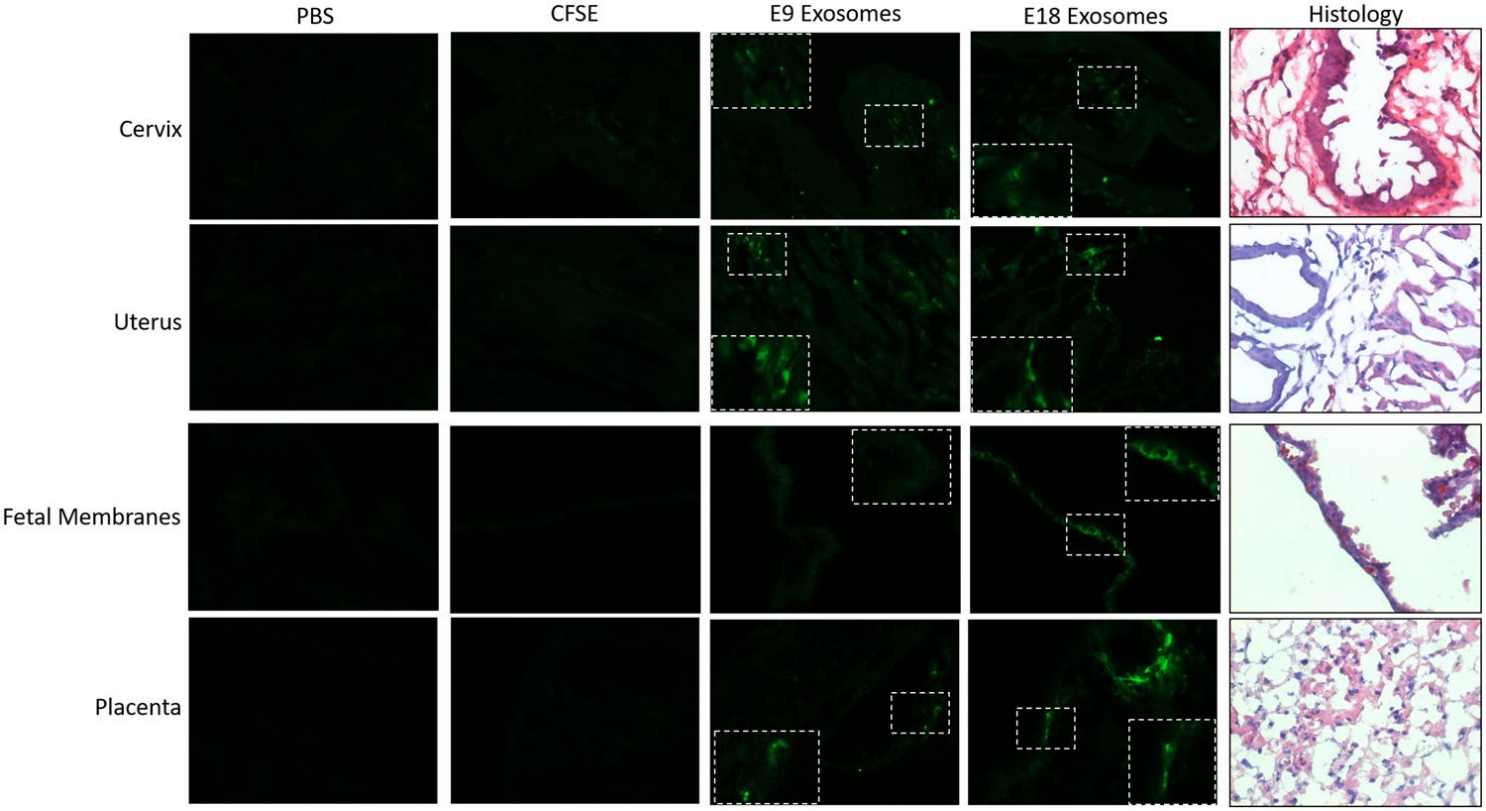
Exosomes injected into pregnant mice traffic to the cervix, uterus, fetal membranes and placenta. Exosomes fluorescently labeled with carboxyfluorescein succinimidyl ester (CFSE), as well as PBS and CFSE in PBS (7.5 µM) were injected into mice on E15. Maternal and fetal tissues were collected and analyzed for fluorescent signal emerging from exosomes. PBS- and CFSE-injected mice show no fluorescent signals, while fluorescent signals from labeled exosomes (E9 and E18; green) can be seen in both maternal and fetal tissues tested. The inset is an enlargement of the tissue area marked by white boxes. Histology panel is representative H&E staining for orientation and localization. All images were taken at 40x magnification.

Exosomes from both E9 and E18 mice were found to reach specific and relevant reproductive tissues. Intraperitoneal injection of E9 and E18 exosomes labeled with CFSE (green fluorescence in Fig. 5) were localized in the maternal cervix. In the cervix, exosomes, regardless of gestation day, were primarily localized to the connective tissue, whereas exosomes were localized to the myometrium in the uterus. On the fetal side, E9 and E18 exosomes were localized to the epithelial layer in the fetal membranes and the labyrinth in the placenta. As expected, PBS-injected mice did not show any green fluorescence in any of the tissues investigated (Fig. 5). CFSE diluted in PBS (7.5 µM) was injected as a vehicle control to show nonspecific dye localization in tissues. As shown in Fig. 5, localization of the dye was not found in maternal or fetal tissues, thus suggesting that the localization data were exosome specific. These results were observed on E17.

#### E18 exosomes promote proinflammatory processes to prepare the cervix and uterus for parturition

**Figure 6:**
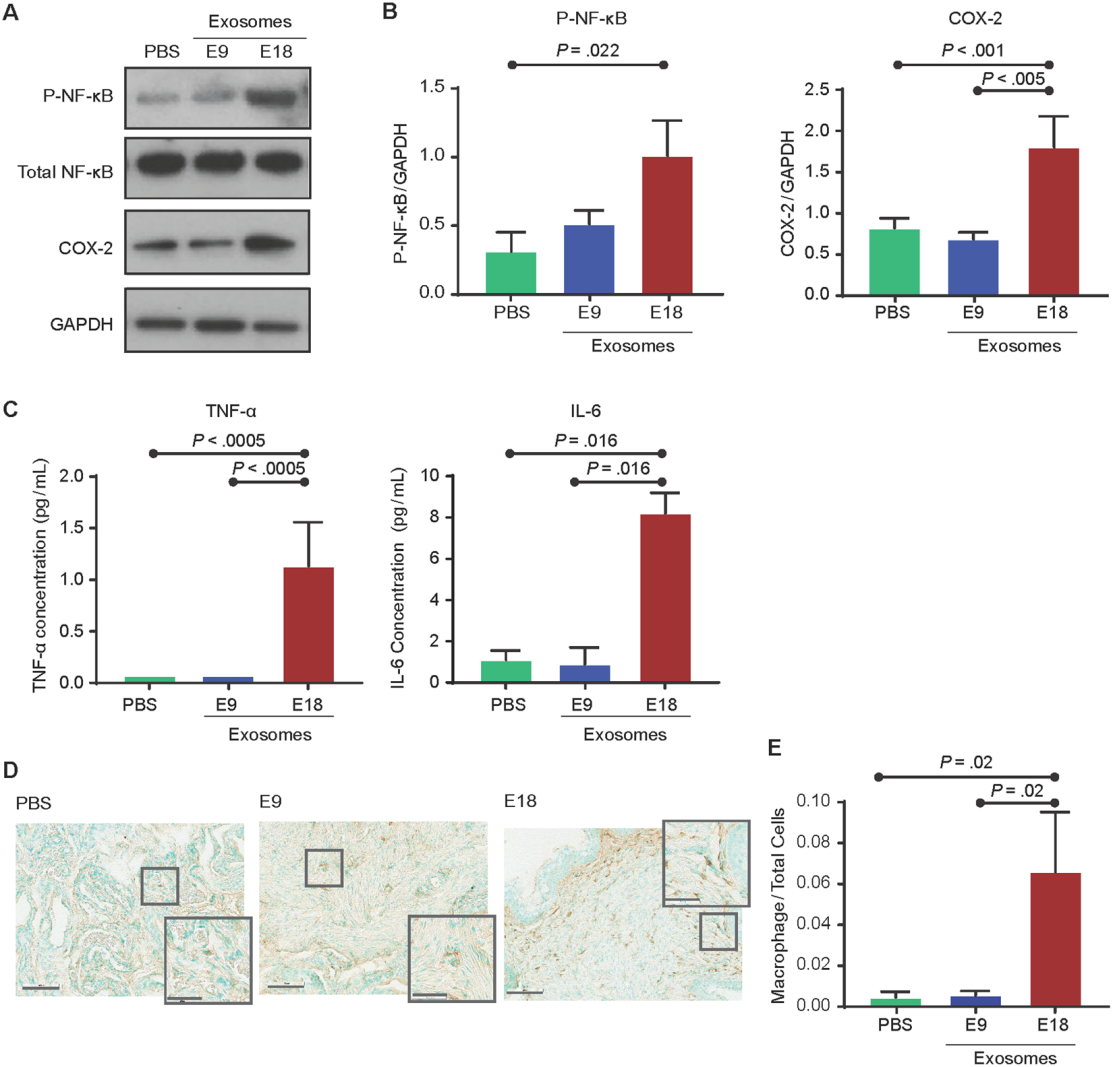
Exosomes injected into pregnant mice induce labor-associated changes in the cervix. (**A–C**) Western blot analysis and densitometry quantitation show a significant increase in the activation of the inflammatory signalers NF-κB (as determined RelA phosphorylation) and COX-2 in E18 exosome-injected mice compared to PBS- and E9 exosome-injected mice. Inflammatory cytokines IL-6 and TNF-α were significantly increased in E18 exosome-injected mice compared to PBS- and E9 exosome-injected mice. Full-length blots are presented in Supplemental Fig. 3. (**D**,**E**) Histology showing that macrophage activation marker F4/80 was significantly increased in E18 exosome injected mouse cervix compared to E9 and PBS control injected mice. Nuclei were stained green by methyl green and macrophages were stained brown. For a semi-quantitative estimation of macrophage activation, total cells and macrophages were counted to determine macrophage to total cell ratio (bar graph).

Based on the effect of day 15–16 treatments with E18 exosomes to induce preterm birth on E18, maternal tissues were analyzed for labor-associated inflammatory changes on E17 (the day prior to preterm birth in this model). In the cervix (Fig. 6A,B), E18 exosome-injected mice had significantly higher NF-κB activation (expressed as arbitrary units) compared to the PBS controls (1.011 ± 0.51 vs. 0.3135 ± 0.22; *P* = 0.022). COX-2 expression in E18-injected mice (1.458 ± 0.22) was also significantly higher than in E9 exosome- and PBS-injected mice (0.6874 ± 0.1637; *P* < 0.005 and 0.8219 ± 0.23, *P* < 0.0001, respectively). Cytokine analysis (Fig. 6C) showed a significant increase in the labor-associated cytokine, IL-6, in E18 exosome-injected mice (8.20 ± 1.72 pg/mL) compared to E9 exosome- and PBS-injected mice (0.89 ± 4.30 pg/mL; *P* = 0.01 and 1.11 ± 0.87 pg/mL; *P* = 0.02, respectively). Similarly, the TNF-α concentration was also significantly higher (*P* < 0.0005) in E18 exosome-injected mice (1.14 ± 0.84 pg/mL) compared to E9 exosome- (not detectable) and PBS- (not detectable) injected mice. Histologic analysis and quantitation of macrophage infiltration and activation in cervix of E18, E9 exosome and PBS injected mice (Fig. 6D,E) showed significantly increased numbers of cells positive for macrophage marker F4/80 in E18-injected (0.066 ± 0.083) mice compared to E9- (0.006 ± 0.007, *P* = 0.02) and PBS- (0.005 ± 0.009, *P* = 0.02) injected mice (Fig. 6E).

**Figure 7:**
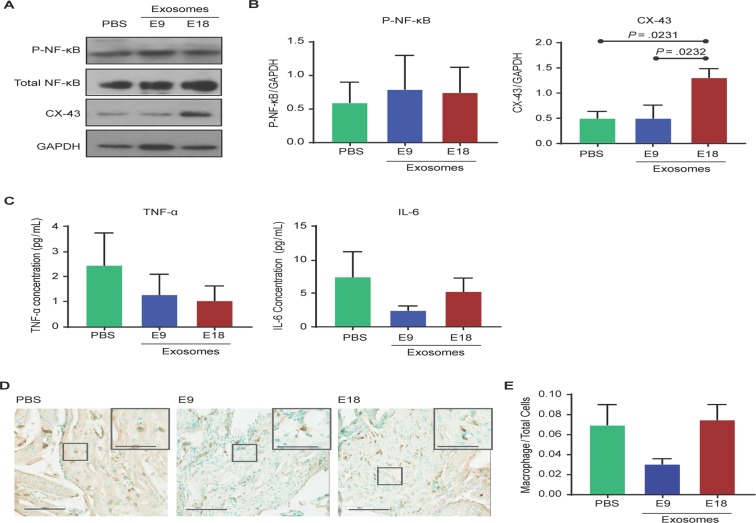
Exosomes injected into pregnant mice induce labor-associated changes in the uterus. (**A–C**) Western blot and densitometry quantitation of NF-κB activation (as determined by RelA phosphorylation) and connexin-43 (CX-43) expression show that NF-κB activation is not significantly different between treatments, while CX-43 expression is significantly increased in E18 exosome-injected mice compared to PBS- or E9 exosome-injected mice. Inflammatory cytokines IL-6 and TNF-α were not significantly increased in E18 exosome-injected mice compared to PBS- and E9 exosome-injected mice. Full-length blots are presented in Supplemental Fig. 4. (**D**,**E**) Histology showing that macrophage activation marker F4/80 was not significantly different regardless of treatment. Nuclei were stained green using methyl green and macrophages were stained brown. Total cells and macrophages were counted to determine macrophage to total cell ratio (bar graph).

In the uterus, E18 exosome-injected mice did not exhibit significantly different NF-κB activation compared to PBS- or E9 exosome-injected mice (Fig. 7A,B). However, there was a significant increase in the expression of CX-43 (Fig. 7A,B) in E18 exosome-injected mice (1.300 ± 0.350) compared to PBS- and E9 exosome-injected mice (0.495 ± 0.230; *P* < 0.05 and 0.496 ± 0.440; *P* < 0.05, respectively). No significant differences were seen in the tissue levels of IL-6 and TNF-α between the various injected groups (Fig. 7C). Histologic analysis and quantitation of macrophage infiltration and activation in uterus of E18, E9 exosome and PBS injected mice (Fig. 7D,E) showed no significant increase in number of cells positive for macrophage marker F4/80 in E18-injected (0.075 ± 0.045) mice compared to E9- (0.031 ± 0.015, *P* = 0.07) and PBS- (0.070 ± 0.061, *P* = 0.10) injected mice.

#### E18 exosomes promote prepartum proinflammatory processes in fetal membranes but not in the placenta

**Figure 8:**
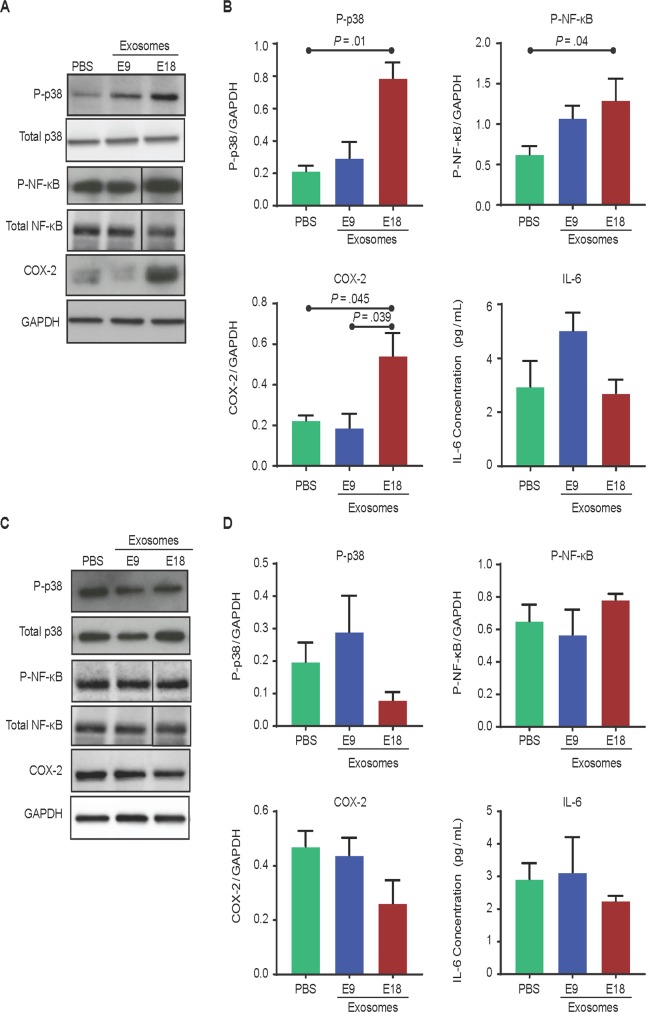
Exosomes injected into pregnant mice traffic to the fetal tissues and induce inflammatory changes in the fetal membranes but not the placenta. (**A–C**) Western blot analysis and densitometry quantitation show significant activation of NF-κB (as determined by RelA phosphorylation), COX-2, and the prosenescence marker p38 MAPK in E18 exosome-injected mice compared to PBS- or E9 exosome-injected mice. No significant difference in proinflammatory cytokine IL-6 levels was observed regardless of the treatment. Full-length blots and corresponding loading controls are presented in Supplemental Fig. 5. (**D**–**F**) Western blot and densitometry analysis of NF-κB activation (as determined by RelA phosphorylation), COX-2, and p38 MAPK expression in the placenta show no significant difference in the activation of the prosenescence marker p38 MAPK and inflammatory markers NF-κB, COX-2, and IL-6 between the treatment and control groups. Full-length blots and corresponding loading controls are presented in Supplemental Fig. 6.

In fetal membranes, western blot analysis of p38 MAPK activation, RelA phosphorylation, and COX-2 expression indicate fetal tissue response to exosomes injected on the maternal side (Fig. 8A). Quantification was performed using densitometry (expressed as arbitrary units) (Fig. 8B). E18 exosome-injected mice had significantly increased p38 MAPK activation (0.787 ± 0.166) compared to E9 exosome- and PBS-injected mice (0.220 ± 0.172; *P* = 0.01 and 0.214 ± 0.057; *P* = 0.003). This supports previous reports that showed that accelerators of fetal membrane senescence via p38 MAPK can cause sterile inflammation and lead to labor and delivery. P-RelA was significantly higher after E18 exosome injection compared to PBS (1.557 ± 0.787 vs. 0.664 ± 0.229; *P* = 0.04); however, although P-RelA was increased in E18 exosome- compared to E9 exosome-injected mice, it was not significant. In addition, COX-2 expression was also higher in E18 exosome-injected mice (0.623 ± 0.258) compared to E9 exosome- and PBS-injected mice (0.359 ± 0.310; *P* = 0.04 and 0.253 ± 0.053; *P* = 0.04, respectively). IL-6 was not different between the treatment groups (Fig. 8C), and TNF-α was not detectable.

**Figure 9:**
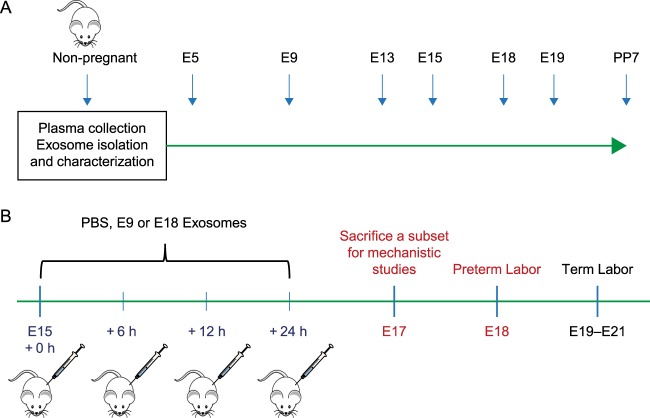
Experimental model: maternal plasma collection and injection timelines in a CD-1 mouse model of pregnancy. (**A**) Experimental Design. Plasma from NP mice and pregnant mice was collected on gestation day (E)5, 9, 13, 15, 18, 19, and PP7 for exosome isolation and characterization. (**B**) Experimental design for PBS and exosome injections to determine the functional role of early (E9), and late gestation (E18) exosomes *in vivo*. PBS, E9 exosomes, or E18 exosomes were injected every 6 h on E15 and once more on E16. Mice were monitored for preterm labor prior to the expected delivery day (E19).

Both E18 and E9 exosomes were localized in the placental tissue (Fig. 6), thus confirming its trafficking to both fetal tissues; however, western blot analysis for P-p38, P-RelA, and COX-2 (Figs 8D and 9E) and cytokine analysis for IL-6 (Fig. 8F) in placental tissue was not significantly different between E18-, E9-, and PBS-injected mice. In addition, TNF-α was not detectable, thus indicating that placental tissue did not respond to exosome-induced inflammation. These data suggest minimal placental changes associated with exosome-induced labor and delivery in this mouse model.

## Discussion

This report tested the hypothesis that exosomes can act as paracrine signals that traffic between maternal and fetal tissues, cause functional inflammatory changes, and induce labor and delivery in a CD-1 mouse model. The 5 major findings of this study were: (1) the injection of E18 exosomes caused preterm birth; (2) by E17, there was evidence of inflammation related to characteristics of maternal cervix remodeling and uterine activation, as well as premature senescence of fetal membranes; (3) the size and shape of maternal plasma exosomes remained constant throughout pregnancy; however, their quantity increased as gestation progressed, with the maximum number seen on E18 (the day before the expected delivery date), before returning to normal 7 days after pregnancy; (4) exosome protein cargo varied at different gestational days, as evidenced by E9 exosome treatments had little or no effects on endpoints of inflammation in reproductive tract tissues; (5) E18 exosome-induced preterm birth was independent of changes in placental function or systemic progesterone withdrawal as plasma levels did not change after treatment. Thus, findings suggest that E18 exosomes provided information that prepares the reproductive tract for parturition at term because when this exosomal cargo is provided on E15–16, preterm birth results.

As seen in this mouse study, human pregnancy is also associated with a progressive increase in maternal plasma exosome concentrations as gestation progresses^47^. Exosomes in the maternal plasma are of both fetal and maternal origin, and the increased concentration throughout gestation is likely contributed to by the growing fetus, the placenta, and fetal membranes^47^. Ongoing studies in our lab (data not shown) suggest that the fetus and fetal tissues (membranes and placenta) contribute approximately 20% of exosomes on E18. The maternal plasma exosome concentration also increases as gestation progresses in mouse pregnancies, peaking on E18 and decreasing postpartum. This is similar to data reported by Salomon *et al*. in human pregnancies and suggests that the days preceding labor and delivery are marked by an increased number of exosomes in the maternal plasma^44^.

Irrespective of the species, inflammation is the mechanistic effector of labor and delivery, and labor inducing inflammation is not systemic but is localized to intrauterine tissues^29,67–70^. Proteomic and bioinformatic analysis determined that exosomal cargo on E18 included proteins linked with the inflammatory response and immune cell migration, which are physiological effectors of labor^54,55,66,71^. An influx of proinflammatory cargo-rich exosomes (E18 exosomes) in uterine tissues, and the biological pathways likely elicited by these cargo contents, eventually cause preterm birth in animals. This is supported by the data reported here, where NF-κB activation and cytokines increased in feto-maternal tissues. Exosomes from E9 are a good control for comparison to show that E18 exosomes specifically induce labor; however, given that E18 exosomes were more inflammatory, it is likely that high levels of these inflammatory exosomes induced labor. The lack of systemic progesterone withdrawal indicates that paracrine mediators can cause labor-associated changes that are independent of endocrine signals. Findings raise the possibility that the quantity of exosomes or specificity of cargo of mature E18 exosomes on critical components in the mechanism for parturition have yet to be examined. Major roles for prostaglandin dehydrogenase and progesterone receptor isoforms in prepartum cervix and uterus have been proposed for parturition^72–74^. Clearly, inflammatory signal carrying exosomes are sufficient to cause labor. These exosomes likely override proposed immunosuppressive effects of progesterone in the cervix and the uterus. In a related study, exosomes from oxidative stress-induced human amnion epithelial cells caused functional progesterone withdrawal (progesterone receptor [PR]A:PRB ratio switch) in human myometrial cells, which is a theorized mechanism associated with human labor^74–76^. Therefore, the possibility that exosomes may also cause endocrine disruption to ensure labor and delivery cannot be excluded.

In mice, cervical ripening and distention begin long before the initiation of labor. In the present study, a greater inflammatory response to E18 exosomes was found in the cervix compared to uterine tissues. This is an expected event before labor and delivery as cervical ripening precedes uterine changes^77,78^. Increased presence of macrophages occur by E17, 2–3 days before birth^79,80^. NF-κB activation likely induces proinflammatory cytokines and an increase in COX-2 in the cervix, thus supporting ripening-associated changes. The CX-43 increase was the only statistically relevant observation in the uterus. NF-κB activation trended in the same direction as seen in the cervix; however, the increase was not sufficient to reach statistical significance. This was likely due to the uterine tissues slowly responding to the changes in the ripening cervix on E17. Activation and infiltration of immune cells was also not significantly different in the uterus. This trends with current literature indicating leukocyte infiltration of the uterus does not increase until active labor^81,82^. The lack of systemic inflammation (no change in plasma IL-6 and TNF-α irrespective of exosome or PBS injection) indicates that inflammation in the cervix and uterus is highly localized and that signals propagate within this region to exclusively modify the environment to prime specific target tissues for labor. The timing of preterm labor is also worthy of discussion. In LPS-treated animals, systemic inflammation (data not shown but reported by many groups^83–85^, caused preterm labor within 24 h of injection. In E18 exosome-treated animals, multiple injections were necessary to compensate for the short circulating lifespan of exosomes and to mimic the consistent influx and concentration of exosomes from both the fetus and the mother as term approaches. The lack of systemic inflammation and the gradual development of inflammation (as observed in E17 tissues) was archetypal of such changes during pregnancy before labor. Hence, the delivery on E18 was as expected in this model.

We expected exosomes to cause changes in both maternal and fetal side. As shown in our results, exosomes (E9 and E18) injected on the maternal side were shown to traffic to the fetal compartment (placenta and fetal membranes). Although no functional changes associated with labor and delivery were seen in the placenta, increased inflammation and activation of prosenescence marker p38 MAPK was observed in mice injected with E18 exosomes. Previous studies showed that fetal membrane senescence is regulated by p38 MAPK in humans^17,24^ and mice^34^. Activation of p38 leads to fetal membrane senescence, a mechanism shown to be associated with the initiation of labor at term and is accompanied by the release of SASP and DAMPs, which may be carried to the maternal tissues by exosomes and support fetal-maternal crosstalk to prepare for labor. Thus, increased p38 MAPK in the fetal membranes on E17 (the day prior to observed preterm labor in this model) may be an important mechanistic contributor to the preterm labor observed in this study.

This study determined the contribution of total plasma exosomes in the initiation of labor; however, the specific or independent contributions by either fetal or maternal exosomes are still unknown. Therefore, it is still not possible to elucidate which paracrine signal (the mother, the fetus, or both) initiates labor. This is primarily due to the lack of fetal-specific exosome markers in mouse models, unlike in humans where exosome-expressed placental alkaline phosphatase is used to differentiate fetal (placental)-derived signals^31,46,48^. Our lab has developed a transgenic mouse model in which fetal exosomes are tagged and can be sorted from maternal blood. Further characterization of these exosomes and similar studies using maternal- and fetal-specific exosomes may determine the requirement of feto-maternal specific signals in the initiation of labor in mice.

The lack of systemic progesterone withdrawal or systemic inflammation supports an independent functional role for exosomes in labor and delivery. In addition, trafficking of exosomes was also observed from the maternal side to the fetal side, where they produced functional changes (i.e., senescence and sterile inflammation) in fetal membranes as expected at this gestational age^34,62^. Exosomal cross talk between mother and the fetus and the functional changes they can cause may be one of the non-endocrine mechanisms that will determine the timing of pregnancy. Unlike pathological stimulants (e.g., LPS, TNF, and IL-1 injections)^86–88^, exosomes are more physiologically representative. Slow development of labor (preterm in this model) associated changes in cervix and uterus (48-h post injection) suggests a gradual, natural, and physiological buildup of exosomes (quantitatively and qualitatively), reflects tissue-level changes exerted by exosomes, and a progressive programing of uterine tissue for labor and delivery. It should be noted that the exosome doses used for the injections (E9 vs. E18) were different. This was done to mimic the systemic exposure experienced on those specific days.

Exosomes, as paracrine signalers, can induce labor-associated change in feto-maternal uterine tissues leading to labor and delivery in the absence of systemic progesterone withdrawal. This is the first study to report exosome characterization throughout murine pregnancy, and the results show that exosomes on the maternal side can cross the placenta to reach the fetal side. In addition, this study suggests a potential role for exosomes in the initiation and progression of labor. Further studies must be performed to determine the maternal and fetal contribution to better understand the communication during pregnancy and specifically during labor and delivery. Human studies on term and preterm parturition are restricted mostly to endocrine or cytokine mediated labor initiation signals. Knowledge of exosome-mediated paracrine signaling of labor-associated changes and induction labor provides new avenues of research on how exosomes may contribute to normal and adverse labor events.

## Methods

### Animal care

Procedures were approved by the Institutional Animal Care and Use Committee (IACUC) at the University of Texas Medical Branch, Galveston. Timed-pregnant CD-1 mice (Charles River Laboratories, Houston, TX) were received on gestational day 4 (E4) or 14 (E14). Mice were housed in a temperature- and humidity-controlled facility with automatically controlled 12:12-h light and dark cycles with regular chow and drinking solution provided ad libitum. Certified personnel and veterinary staff provided regular maintenance and animal care according to IACUC guidelines. The animals were sacrificed by CO_2_ inhalation according to the IACUC and American Veterinary Medical Association guidelines.

### Maternal plasma collection

Maternal blood (0.5–1.0 mL) from timed-pregnant CD-1 mice was collected by cardiac puncture in tubes that contained EDTA (Becton Dickinson, Franklin Lakes, NJ) on gestation day (E) 5, 9, 13, 15, 18, and 19 and on postpartum day 7 (Fig. 9A), as well as from nonpregnant mice. Mice were sacrificed by CO_2_ asphyxiation. Plasma was harvested after centrifugation (2000 × g for 10 min at 4 °C) and stored at −80 °C.

### Maternal plasma exosome isolation

Exosomes from maternal plasma were isolated as described previously with modifications^89–91^. Briefly, plasma samples were thawed on ice, diluted in 1.0 mL of cold 1x phosphate buffered saline (PBS), and centrifuged at 2000 × g for 10 min at 4 °C. Supernatants were transferred to clean microcentrifuge tubes then filtered through Nalgene™ Syringe Prefilter Plus (Thermo Fisher, Waltham, MA). After filtration, samples were centrifuged at 10,000 × g for 30 min. The supernatant was transferred to ultracentrifuge tubes and centrifuged at 100,000 × g for an additional 2 h. The supernatant was subsequently discarded, and the pellet was resuspended in 100 μL of cold 1x PBS. The final pellet was passed through an Exo-spin™ column (Cell Guidance Systems, St. Louis, MO) following the manufacturer’s instructions. Samples were aliquoted and stored at −80 °C.

### Determination of exosome shape using cryo-electron microscopy

To determine the shape of exosomes in maternal plasma, 3 μL of prepared exosome suspension was pipetted onto a copper grid with quantifoil support film (QUANTIFOIL, Germany). The support film was patterned with a regular array of circular holes. When the excess liquid was blotted away from the grid, a 60–120 nm-thick film of sample suspension remained in these holes. The grid was then plunged into a small crucible of liquid ethane that was cooled to its melting point by liquid nitrogen. In the liquid ethane, the sample suspension was cooled at over 10,000 degrees/s, solidifying the water in an amorphous state. This “vitrification” process preserves the exosomes in their native state without distorting their geometry by crystallization and density change^41^. The vitrified sample on the grid was then placed in a Gatan 626 specimen holder (Gatan, Pleasanton, CA), which was placed in a JEOL 2100 TEM (JEOL, Osaka, Japan). The samples were imaged with a 200 kV electron beam from a LaB6 emission source, and images were recorded on a Gatan US4000 CCD camera. Images were captured at 15,000–30,000 magnification.

### Determination of exosome size and quantification

The size distribution and concentration of exosomes were determined using the Nanosight NS300 (Malvern Instruments, Worcestershire, UK). Samples were diluted (1:500) in distilled water and run following the manufacturer’s instructions. A camera level of 12 and automatic functions were used for all postacquisition settings, except for the detection threshold which was fixed at seven. The camera focus was adjusted to make the particles appear as sharp dots. Three 30 s videos were recorded for each sample using the script control function.

### Flow cytometry analysis for exosome markers

For flow cytometry analysis of exosome tetraspanin marker CD63, samples were prepared using the ExoFlow kit (System Biosciences, Mountain View, CA) protocol with modifications. Briefly, 9.1 μm streptavidin-coated beads were washed and incubated with biotinylated anti-CD63 (clone MX-49.129.5, Novus Biologicals, Littleton, CO) for 2 h on ice, flicking intermittently to mix. The beads were washed and resuspended in 200 μL of bead wash buffer prior to overnight incubation (4 °C) with 100 μL of exosomes. The following day, samples were washed and stained using the Exo-FITC exosome stain according to the manufacturer’s instructions, before being run on the Cytoflex flow cytometer (Beckman Coulter, Brea, CA). Negative controls were beads incubated with antibody but without exosomes, and these controls were used for gating according to the manufacturer’s instructions. Data analysis based on the fluorescein isothiocyanate (FITC) signal shift was performed using Cytexpert (Beckman Coulter).

### Proteomic analysis of maternal plasma exosomes by mass spectrometry

#### Exosome protein clean-up and digestion

The protein profile of maternal plasma exosomes was established by liquid chromatography (LC) and mass spectrometry (MS). Exosomes in 1x PBS (12.5 μL) were lysed in 12.5 μL of 10% SDS in 0.1 M TEAB by sonicating on ice for 10 min. A total of 1 μL of 0.25 M TCEP was added to each tube to attain a final concentration of 0.01 M, and samples were incubated at 55 °C for 1 h. The samples were allowed to cool before 1 μL of 20 mM iodoacetamide was added to each tube and the samples were incubated for 45 min in the dark. The samples were subsequently transferred to tubes containing 8 M dry urea and vortexed. A total of 2.7 μL of 12% phosphoric acid was added, and 165 μL of S-Trap buffer (90% MeOH, 100 mM TEAB, pH 7.5) was added to the acidified lysate. The lysate was added to the S-Trap microcolumn (Protifi, Huntington, NY) and centrifuged at 4000 × g for 2 min. The flow-through was discarded and the column was washed with 150 μL of S-Trap buffer before being centrifuged at 4000 × g for 2 min. Columns were transferred to new tubes and incubated with 16 ng/mL of trypsin for 2 h at 47 °C. Proteins were eluted by centrifugation at 4000 × g for 2 min with 50 mM TEAB, 0.2% formic acid (FA), 50% acetonitrile (ACN)/0.2% formic acid, and finally 80% acetonitrile/0.1% formic acid. After the elutions, the final elute was dried.

#### LC-MS/MS analysis of plasma exosomes

Nano LC-MS/MS data acquisition was performed on the OrbiTrap Fusion mass spectrometer system (Thermo Fisher Scientific, San Jose, CA) coupled to a Dionex Ultimat 3000 nano-HPLC with a 40-well standard tray autosampler. The sample (5 μL) was injected onto a trap column (300 um i.d. × 5 mm, C18 PepMap 100) and then a C18 reverse-phase nano LC column (100 75 um × 25 cm, Acclaim PepMap), which was heated to 50 °C in a chamber. The loading pump flow rate was set to 8 μL/min. The nano pump flow rate was set to 400 nL/min with a 130-min LC gradient, where the mobile phases were A (99.9% water and 0.1% FA) and B (99.9% ACN and 0.1% FA). The gradient was 0–5 min at 2% of B, 6–100 min at 4–32% of B, an increase to 50% at 108 min, 5 min of 90% B wash, and 15 min equilibrium. Mass spectrometer parameters included tip spray voltage at +2.2 kV, sweep gas 1, and an ion-transfer tube temperature of 275 °C. FTMS mode for the MS full scan from 350–1500 Da was used for the acquisition of precursor ions (resolution 120,000), ITMS top-speed (3 s) mode was used for MS/MS, and MS/MS was accomplished via HCD with collision energy of 32%.

All MS/MS samples were analyzed using Proteome Discoverer 1.4.1.14, which was set up to search UniProt-mouse.fasta (downloaded April 2016). Proteome Discoverer was searched using a fragment ion mass tolerance of 0.60 Da and a parent ion tolerance of 10.0 PPM. Peptide charges considered were +2, +3 and +4. Scaffold (version 4.8.4, Proteome Software Inc., Portland, OR) was used to validate MS/MS-based peptide and protein identifications. Peptide identifications were accepted if they could be established at >99.0% probability to achieve a false discovery rate (FDR) <1.0% by the Peptide Prophet algorithm^92,93^ with Scaffold delta-mass correction, if they could be established at >99.0% probability and contained at least two identified peptides. Proteins that contained similar peptides and could not be differentiated based on MS/MS analysis alone were grouped to satisfy the principles of parsimony. Proteins were annotated with gene ontology (GO) terms from NCBI (downloaded on September 1, 2017)^94^. Proteins were normalized to nonpregnant samples (median area under curve) and considered significantly different when *P* < 0.01 and the fold change was ±1.5.

### Ingenuity pathway analysis (IPA) of identified proteins

Pathway enrichment analyses were performed with IPA (Qiagen, Hilden, Germany) using Fisher’s exact test. IPA was performed to identify canonical pathways, biological functions, and protein networks. Heatmap and hierarchical cluster analyses (ClusterMaker, open source) were used to demonstrate the expression patterns of the differentially-expressed proteins and pathways based on fold change and Z-scores. Significantly enriched pathways for the proteins and scenarios were identified using *P* < 0.01.

### Injection of E9 and E18 exosomes to determine trafficking and labor-associated functional changes

To test the functional effects of exosomes, maternal plasma exosomes from E18, typically the day prior to birth on E19.5) in this strain, and E9 (early gestation control) were used. Exosomes from these days were chosen based on data showing that E9 exosomes have high levels of anti-inflammatory proteins and E18 have maximum levels of proinflammatory proteins (see results section). On E15, mice were injected three times at 6-h intervals and 12-h later on E16 with exosomes (3.33 × 10^10^ or −9.16 × 10^10^ in 250 μL PBS, Fig. 9B) of PBS. Concentrations were based on physiological levels during normal pregnancy (see Results section) and treatment on E15 was chosen based upon other models of inflammation-induced preterm birth^95–97^ Multiple doses of exosomes were administered to sustain systemic concentrations because exosome half-life is relatively short^98,99^ and a single dose does not affect pregnancy duration. Mice were monitored for preterm birth, which was defined as delivery on or before E18, compared to the expected delivery date of E19–E21 in controls. To further understand the functional effects of exosomes, tissues were obtained from four mice per treatment group on E17 as described above, fixed in 10% neutral-buffered formalin or flash frozen in liquid nitrogen.

Infection-induced preterm labor was used to confirm preterm labor in this model by injecting 100 µg of LPS serotype O55:B5 (in 100 µL PBS) intraperitoneally into pregnant mice on E15. This serotype and dose are commonly used in LPS-induced preterm birth models^70,100,101^. This model was used as a true positive control for our animal models of preterm birth to show that preterm birth was indeed possible at an early gestational day and what we expect to observe with exosomes are a physiologic development of labor associated changes induced by exosomes.

### Fluorescent labeling of exosomes for determining *in vivo* trafficking and localization

Maternal plasma exosomes from E9 and E18 were isolated and labeled with carboxyfluorescein succinimidyl ester (CFSE) by resuspending them in 100 μL of 7.5 μM CFSE. Exosomes were incubated at 37 °C for 30 min before being diluted in PBS containing 5% BSA. Exosomes were run through the Exo-spin™ column (as described above) to remove excess CFSE.

### Immunofluorescent imaging for exosome trafficking

Tissue samples collected in 10% neutral-buffered formalin were stored overnight (4 °C) before being washed twice with 1x PBS and transferred to a 15% sucrose solution overnight (4 °C). Samples were then transferred to 30% sucrose and stored at 4 °C until they were embedded in optimal cutting temperature (OCT) and cut into 5 μm sections. Sections were washed twice in water and treated with FITC Block (Abcam) for 15 min at room temperature. After a further two washes in water, tissues were mounted using Mowiol 4–88 mounting medium. Exosomes were visualized using the Olympus BX43 fluorescent microscope (Olympus, Tokyo, Japan) at 40x magnification, and images were captured using Q Capture Pro software. Brightness, contrast, and smoothing were applied to the entire image using FIJI (open source). Routine hematoxylin and eosin (H&E) histochemical staining was performed on the serial sections to ensure proper histology of tissues used.

### Enzyme-linked immunosorbent assay (ELISA) for progesterone levels in maternal plasma

Maternal plasma samples were collected on E17 and analyzed for progesterone levels. Plasma progesterone levels were determined using the Mouse Progesterone ELISA kit (Biotang Inc., Lexington, MA) following the manufacturer’s instructions. Concentrations below the assay detection limits (0.5 ng/mL) were considered as one-tenth of each value

### Immunohistochemistry for macrophage infiltration and activation

To determine that exosomes can cause inflammatory changes in maternal gestational tissues, cervix and uterus were fixed in 10% neutral buffered formalin for 24 h at 4 °C overnight before paraffinization, sectioning (10 μm), and processing as previously described^78^. Sections were stained with the F4/80 antibody to visualize mature macrophages (1:800 BM8; BMA Biomedicals, Switzerland) and counterstained with a 1% methyl green solution to identify cell nuclei. Macrophages were clearly identified by dark brown stain against low background and in association with a methyl green counterstained cell nucleus.

### Western blot analysis

#### Western blot analysis for exosome markers

Exosomes in PBS were lysed by the addition of 10x radioimmunoprecipitation assay (RIPA) lysis buffer (50 mM Tris pH 8.0, 150 mM NaCl, 1% Triton X-100, 1.0 mM EDTA pH 8.0, and 0.1% SDS) supplemented with protease and phosphatase inhibitor cocktails and PMSF. Western blot samples were subsequently processed as described previously^35,38,42^. The anti-mouse antibodies used included exosome markers CD9 (1:400; Abcam, Cambridge, United Kingdom), CD81 (1:500; Cell Signaling, Beverly, MA), and Alix (1:1000; Abcam).

#### Western blot analysis of fetal and maternal tissues

Tissues flash frozen in liquid nitrogen were homogenized in RIPA buffer supplemented with protease and phosphatase inhibitors using a bullet blender (Next Advance, Averill Park, NY) as described previously^34^. Blots were incubated overnight with primary antibodies against total NF-κB (1:1000; Cell Signaling, Danvers, MA), phosphorylated (P)-NF-κB (1:400; P-RelA; Abcam), total p38 MAPK (1:1000; Cell Signaling), P-p38 MAPK (1:400; Cell Signaling), COX-2 (1:800; Abcam), or connexin-43 (1:3000; Abcam). Samples for the same experiment were run on the same gel for a given marker to avoid interassay variability between blots. The blots were all reprobed with antibodies to GAPDH (1:1000; Santa Cruz Biotechnology, Dallas, TX), and all proteins were normalized to GAPDH prior to densitometry analysis. Semi-quantitated data on western blots (based on densitometry readings) are expressed in arbitrary units in the result sections.

### Luminex assay to determine inflammatory markers in tissue and plasma

Plasma and tissues collected from mice on E17 were assayed for IL-6 and TNF-α (n = 4 per group) using MILLIPLEX Mouse Cytokine Panel 1 (Millipore) following the manufacturer’s protocol. For tissues, equal amounts of protein (25 μg) were loaded into each well. Standard curves were developed using duplicate samples of known-quantity recombinant proteins that were provided by the manufacturer. Sample concentrations were determined by relating the absorbance of the samples to the standard curve using linear regression analysis. Concentrations below the assay detection limits (IL-6 = 1.1 pg/mL; TNF-α = 2.3 pg/mL) were considered as one-tenth of each value.

### Statistical analysis

Western blot (n = 4), bioplex and ELISA (n = 4), macrophage quantitation (n = 3) and exosome size and concentration (n = 8) data were analyzed using a one-way ANOVA with Tukey’s post hoc test using GraphPad Prism (GraphPad, San Diego, CA). *P* < 0.05 was considered significant. Proteomic comparison between all gestation days (n = 3) was analyzed using the Kruskal-Wallis test with Benjamini-Hochberg correction using Scaffold 4 Viewer. Comparison between two gestation days was analyzed using the nonparametric Mann-Whitney test with Benjamini-Hochberg correction using Scaffold 4 Viewer. Adjusted *P* value (q value) <0.01 was considered significant.

A post hoc power analysis was performed using G*Power^102^ based on group means, standard deviation, and effect size (f = 1.1 for western blot and cytokine data, 0.81 for progesterone and exosome size and concentration, and 0.93 for preterm birth rates). This analysis revealed that the study had >80% power for the ANOVA to detect differences between groups at a 0.05 significance level.

## Supplementary information

Supplemental Tables and Figures

## Data Availability

The datasets generated during and/or analyzed during the current study are available from the corresponding author on reasonable request.
