# Supplementary material for: Exosomes Cause Preterm Birth in Mice: Evidence for Paracrine Signaling in Pregnancy
Source: Sci Rep. 2019 Jan 24;9:608. doi: 10.1038/s41598-018-37002-x (PMC6345869; doi:10.1038/s41598-018-37002-x)

Exosomes Cause Preterm Birth in Mice: Evidence for Paracrine Signaling in Pregnancy

Samantha Sheller-Miller[1,2], Jayshil Trivedi, MS,[1] Steven M. Yellon, PhD[3], Ramkumar Menon, MS, PhD[1*]

Supplemental Materials

Table S1. Exosome Size and Concentration throughout Gestation.

Gestation Day	Mean Size	Mean Concentration	Average Number of Pups	Adjusted for Number of Pups
Nonpregnant	140.9 (±11.4)	1.24×10^{11} (± 2.41×10^{10})	N/A	N/A
E5	146.0 (±15.0)	1.52×10^{11} (±9.03×10^{10})	N/A	N/A
E9	136.1 (±8.9)	1.75×10^{11} (±4.48×10^{10})	12 (± 3)	1.65×10^{10} (±7.74×10^{9})
E13	157.9 (±19.6)	2.53×10^{11} (±1.27×10^{11})	13 (± 2)	1.94×10^{10} (±6.99×10^{9})
E15	131.3 (±12.4)	2.97×10^{11} (±1.03×10^{11})	10 (± 4)	2.87×10^{10} (±1.56×10^{10})
E18	148.6 (±17.2)	3.66×10^{11} (±8.31×10^{10})	12 (± 3)	3.11×10^{10} (±9.11×10^{9})
E19	155.1 (±29.8)	3.13×10^{11} (±1.47×10^{10})	12 (± 2)	2.81×10^{10} (±1.73×10^{10})
PP7	145.7 (±18.6)	1.39×10^{11} (±4.60×10^{10})	N/A	N/A

Supplemental Table 2: Exosome concentration throughout gestation: Tukey's multiple comparisons

Gestation Days	*P* value
Nonpregnant vs. E5	0.500
Nonpregnant vs. E9	0.478
Nonpregnant vs. E13	0.103
Nonpregnant vs. E15	0.031
Nonpregnant vs. E18	<0.0001
Nonpregnant vs. E19	0.007
Nonpregnant vs.PP7	0.500
E5 vs. E9	0.500
E5 vs. E13	0.253
E5 vs. E15	0.095
E5 vs. E18	<0.0001
E5 vs. E19	0.029
E5 vs. PP7	0.500
E9 vs. E13	0.274
E9 vs. E15	0.092
E9 vs. E18	<0.0001
E9 vs. E19	0.017
E9 vs. PP7	0.497
E13 vs. E15	0.494
E13 vs. E18	0.029
E13 vs. E19	0.446
E13 vs. PP7	0.178
E15 vs. E18	0.383
E15 vs. E19	0.500
E15 vs. PP7	0.059
E18 vs. E19	0.433
E18 vs. PP7	<0.0001
E19 vs. PP7	0.016

Table S3. Top 3 canonical pathways identified with IPA

Acute Phase Response Signaling				
Gestation Day	-Log (q-value)	Z-score	# Molecules Identified	Molecules
E5	18.387	0	13	ITIH3,AMBP,SERPINA3,SAA2-SAA4,FGG,SERPINF2,F2,ALB,ITIH4,CRP,FGB,SERPINA1,FGA
E9	11.032	-1.89	10	KLKB1,ALB,APOA2,SERPINA3,FGB,FGA,A2M,FGG,RBP4,AGT
E13	12.645	0	10	SERPING1,HP,APOA1,APOA2,CRP,SERPINA3,FGB,A2M,FGG,C5
E15	14.62	1.89	13	TTR,ITIH3,APOA2,VWF,SERPINA3,FGG,F2,PLG,C4A/C4B,ALB,ITIH4,SERPINA1,A2M
E18	20.202	1.414	18	APOH,C1S,APOA2,SERPINF1,VWF,SAA2-SAA4,F2,SERPIND1,PLG,C4A/C4B,TF,ITIH4,CRP,SERPINA1,FGB,LBP,A2M,AGT
E19	8.965	1.342	9	ITIH4,APOA2,SERPINF1,VWF,SERPINA3,SERPINA1,FGB,A2M,FGG
PP7	7.272	-2	7	HP,APOA2,AHSG,SERPINA3,SERPINA1,A2M,RBP4
**Liver X Receptor/Retinoid X Receptor (LXR/RXR)**				
Gestation Day	-Log (q-value)	Z-score	Molecules Identified	Molecules
E5	16.345	-2.111	11	KNG1,APOE,ALB,APOB,LCAT,ITIH4,AMBP,SERPINA1,FGA,GC,SERPINF2
E9	9.29	0.707	8	KNG1,ALB,APOB,APOA2,VTN,FGA,RBP4,AGT
E13	2.922	0	3	APOA1,APOA2,GC
E15	10.089	1	9	C4A/C4B,PON1,TTR,ALB,ITIH4,APOA2,VTN,SERPINA1,A1BG
E18	17.898	2.324	15	KNG1,APOB,APOH,APOA2,VTN,SERPINF1,C4A/C4B,PON1,LCAT,TF,ITIH4,SERPINA1,LBP,CLU,AGT
E19	7.324	0.378	7	KNG1,PON1,ITIH4,APOA2,SERPINF1,SERPINA1,A1BG
PP7	5.311	0.447	5	APOC4,APOA2,AHSG,SERPINA1,RBP4
**Coagulation System**				
Gestation Day	-Log (q-value)	Z-score	Molecules Identified	Molecules
E5	12.962	1.89	7	KNG1,SERPINA1,FGB,FGA,FGG,F2,SERPINF2
E9	9.624	-1.633	6	KNG1,KLKB1,FGB,FGA,A2M,FGG
E13	4.521	0	3	FGB,A2M,FGG

E15	15.208	1	9	C4A/C4B,PON1,TTR,ALB,ITIH4,APOA2,VTN,SERPINA1,A1BG
E18	15.957	0.632	10	PLG,KNG1,F5,VWF,SERPINA1,FGB,F7,A2M,F2,SERPIND1
E19	9.237	0	6	KNG1,VWF,SERPINA1,FGB,A2M,FGG
PP7	4.292	0	3	SERPINA1,F13B,A2M

Table S4. Proteins with Log_2 (Fold Change) of ± 0.6 and –Log(q-value) >2.0 for Volcano Plots

Late Gestation (E18) vs Early Gestation (E5)		
**Protein Name**	**log_2 (Fold change)**	**-Log (q-value)**
Serotransferrin	1.38	3.59
Ceruloplasmin	2.30	4.00
Alpha-2-macroglobulin-P	2.76	4.00
Complement C4-B	1.47	4.00
Complement factor H	1.89	4.00
Plasminogen	1.63	4.00
Inter alpha-trypsin inhibitor, heavy chain 4	4.21	4.00
Apolipoprotein B-100	3.00	4.00
Hemopexin	1.46	3.80
Vitamin D-binding protein	1.73	3.80
Leukemia inhibitory factor receptor	4.78	4.00
Corticosteroid-binding globulin	2.96	2.43
Inter-alpha-trypsin inhibitor heavy chain H1	1.74	4.00
Protein Gm20547	1.49	4.00
Prothrombin	2.66	4.00
Gelsolin	1.16	4.00
Carboxypeptidase N subunit 2	1.19	4.00
Inhibitor of carbonic anhydrase	0.99	2.72
Haptoglobin	3.24	2.54
Mannan-binding lectin serine protease 1	1.04	2.00
Apolipoprotein A-IV	1.12	3.00
Beta-2-glycoprotein 1	1.86	2.62
Clusterin	1.30	2.74
Prolow-density lipoprotein receptor-related protein 1	1.78	2.16
Alpha-2-antiplasmin	1.72	2.96
Coagulation factor V	2.88	3.96
Vitronectin	2.36	3.47
Afamin	1.76	4.00
Insulin-like growth factor-binding protein complex acid labile subunit	1.33	2.62
Fetuin-B	1.62	2.29
von Willebrand factor	2.47	2.15
Mannan-binding lectin serine protease 2	1.33	2.11
Sulfhydryl oxidase 1	1.38	2.34
Plasma protease C1 inhibitor	1.85	2.92
Hemoglobin subunit beta-2	-2.84	3.60

Alpha-amylase 1	2.83	2.57
Extracellular matrix protein 1	2.38	3.49
Heparin cofactor 2	2.33	2.46
Tenascin	2.04	2.42
Proteasome subunit alpha type-7	-2.45	4.00
Serine protease inhibitor A3N	2.88	2.44
Zinc-alpha-2-glycoprotein	2.32	2.96
Hepatocyte growth factor activator	4.31	2.00
Carbonic anhydrase 2	-2.19	2.54
Proteasome subunit alpha type-2	-2.36	2.15
Proteasome subunit beta type-2	-1.94	2.05
Proteasome subunit alpha type-3	-2.18	2.62
Catalase	-3.30	2.77
Vascular non-inflammatory molecule 3	2.21	2.62
Complement C1q subcomponent subunit C	2.97	2.37
Proteasome subunit beta type-5	-2.44	3.31
Hyaluronan-binding protein 2	1.65	2.62
Phosphatidylcholine-sterol acyltransferase	2.15	2.19
Aspartyl aminopeptidase	-2.14	3.40
Secreted phosphoprotein 24	1.99	2.72
Ig kappa chain V-VI region XRPC 44	-2.75	2.52
Beta-2-microglobulin	1.40	2.41
**Late Gestation (E18) vs Mid Gestation (E9)**		
**Protein Name**	**$\log_2$ (Fold change)**	**-Log (q-value)**
Fibrinogen alpha chain	1.87	2.01
Complement component C8 beta chain	-0.95	2.07
Mannan-binding lectin serine protease 2	1.26	2.19
Coagulation factor V	1.56	2.39
Heparin cofactor 2	0.85	2.42
Complement C1q subcomponent subunit A	3.56	2.44
Pigment epithelium-derived factor	2.21	2.68
Protein Ighg3 (Fragment)	-2.64	2.77
Complement C1q subcomponent subunit C	4.60	2.77
Hyaluronan-binding protein 2	1.19	2.77
Plasminogen	0.65	2.80
Vascular non-inflammatory molecule 3	2.47	2.89
Complement C1q subcomponent subunit B	3.82	2.96
Complement C1r-A subcomponent	2.52	3.02
Alpha-amylase 1	3.02	3.19
Alpha globin 1	-1.75	3.22

Mannan-binding lectin serine protease 1	1.68	3.38
Hemoglobin subunit beta-2	-2.47	3.42
Thrombospondin-1	1.41	3.64
von Willebrand factor	3.23	3.68
Fibrinogen beta chain	4.24	4.00
Fibrinogen gamma chain	4.69	4.00
Ceruloplasmin	1.58	4.00
Alpha-2-macroglobulin-P	-1.09	4.00
Complement C4-B	1.50	4.00
Complement factor H	1.15	4.00
Inter alpha-trypsin inhibitor, heavy chain 4	1.88	4.00
Inter-alpha-trypsin inhibitor heavy chain H1	1.12	4.00
Prothrombin	1.62	4.00
Prolow-density lipoprotein receptor-related protein 1	1.45	4.00
78 kDa glucose-regulated protein	1.41	4.00
**E18 (Term not in Labor) vs E19 (Term in Labor)**		
**Protein Name**	**$\log_2$ (Fold change)**	**-Log (q-value)**
Alpha-2-macroglobulin-P	-0.95	4.00
Complement factor H	0.80	2.30
Inter alpha-trypsin inhibitor, heavy chain 4	1.49	4.00
Apolipoprotein B-100	0.78	4.00
Hemopexin	1.08	2.21
Inter-alpha-trypsin inhibitor heavy chain H1	0.77	2.82
Prothrombin	0.73	3.41
Apolipoprotein A-IV	1.08	2.96
Murinoglobulin-2	3.51	3.38
Carboxypeptidase N catalytic chain	1.28	2.21
Coagulation factor V	1.38	4.00
H-2 class I histocompatibility antigen, Q10 alpha chain	1.78	3.59
Insulin-like growth factor-binding protein complex acid labile subunit	1.55	3.96
Ig gamma-2A chain C region, membrane-bound form	2.44	2.06
von Willebrand factor	-1.09	3.85
Epidermal growth factor receptor	0.86	2.08
Complement C1q subcomponent subunit B	1.90	3.00
Hepatocyte growth factor activator	1.28	2.15
Vascular non-inflammatory molecule 3	1.20	2.41

Supplemental Figure 1: The injection of E18 exosomes causes preterm birth. Mice were intraperitoneally injected with either PBS, E9 exosomes or E18 exosomes on E15 every 6 hours and again on E16 then monitored for preterm labor.
A: Representative photograph of a nonlaboring pregnant mouse on E18 has a full uterus.
B: Representative photograph of a preterm delivering mouse. After delivery of at least one pup, the mouse was euthanized, and the uterus was exposed. The fetuses contained on the right side of the uterus have been delivered while the left side still contained fetuses.

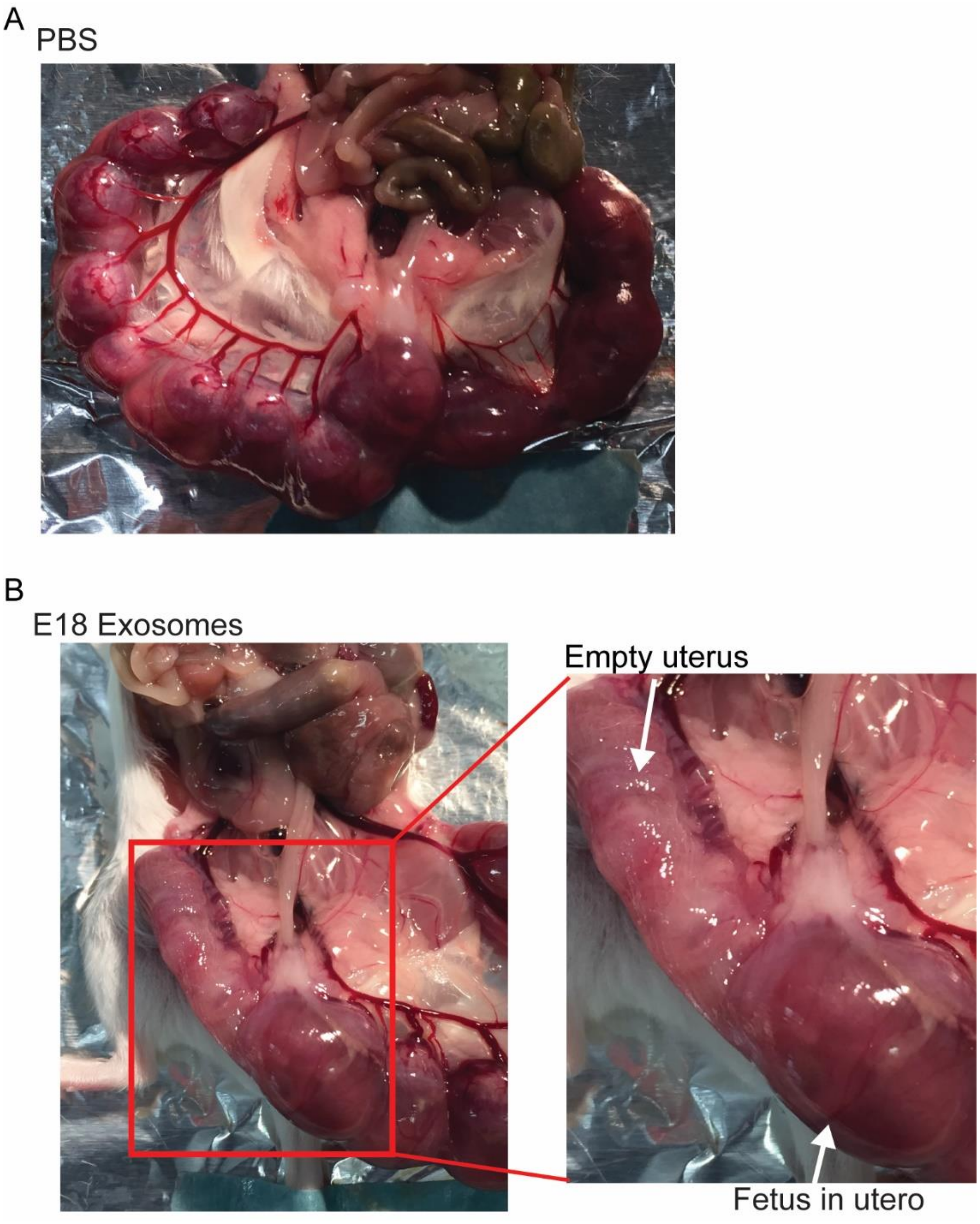

Supplemental Figure 2: Full length blots of exosome markers ALIX, CD9 and CD81.
A: Complete blots for exosome markers.
B: Colormetric image of exosome marker blots to identify molecular weight markers.

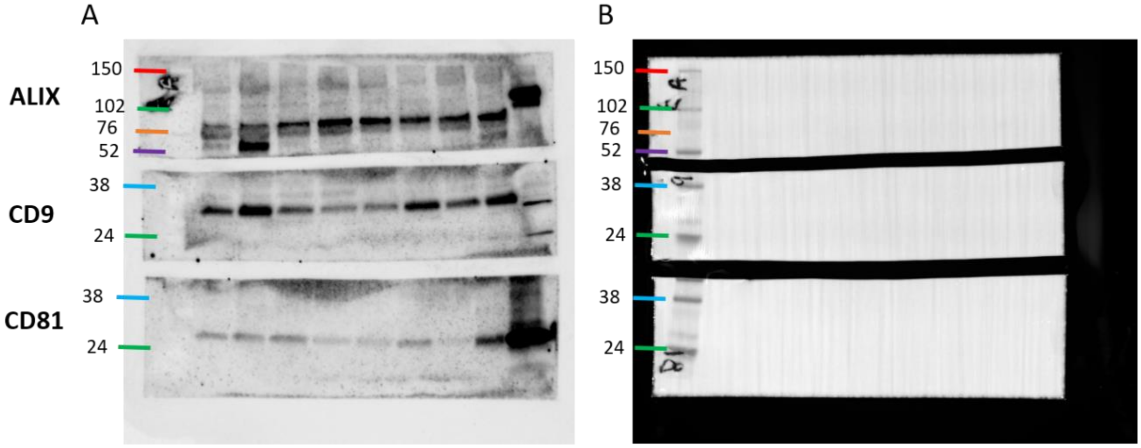

Supplemental Figure 3: **Full length blots of cervical tissues**
Complete blots for p-NF-κB, total NF-κB, COX-2 and GAPDH (loading control). Boxed bands are those used for publication.

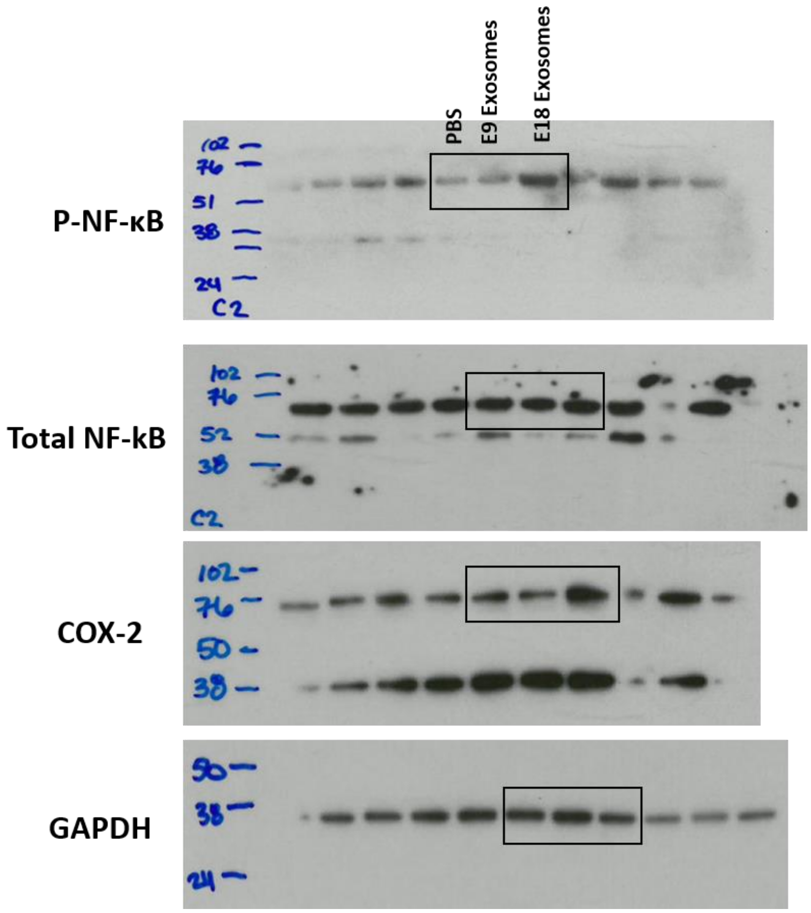

Supplemental Figure 4: **Full length blots of uterine tissues.**
A: Complete blots for p-NF-κB, total NF-κB and GAPDH (loading control). Boxed bands are those used for publication.
B: Complete blots for CX-43 and GAPDH (loading control). Boxed bands are those used for publication.

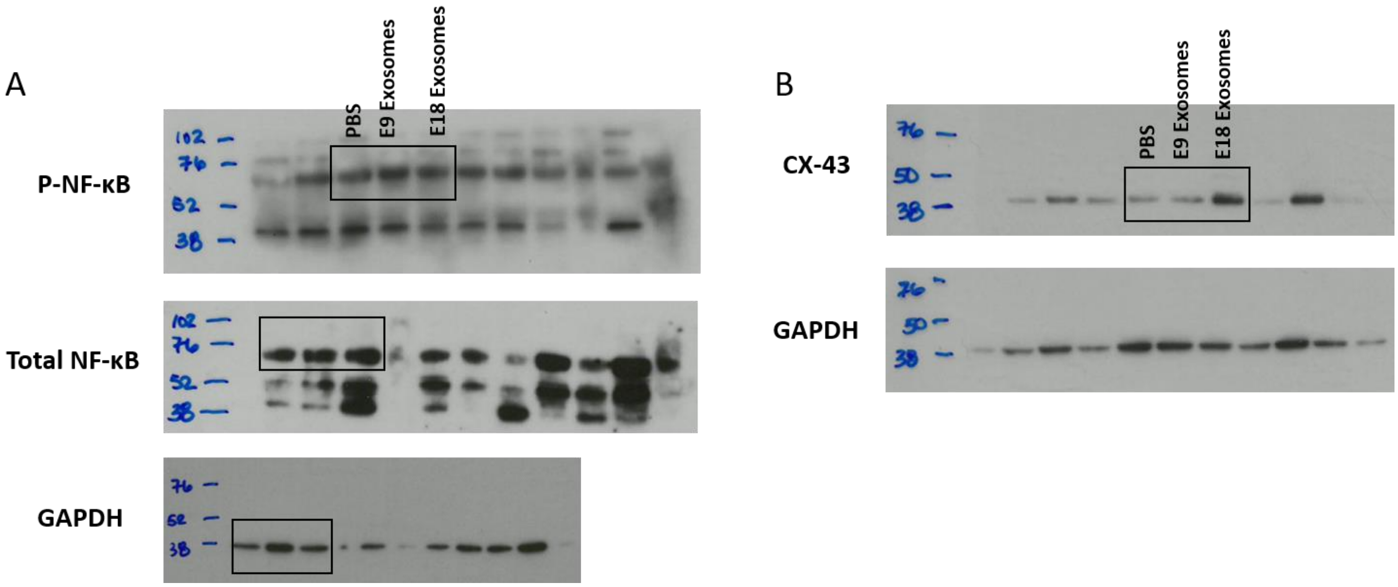

Supplemental Figure 5: **Full length blots of fetal membrane tissues.**
A: Complete blots for p-NF-κB, total NF-κB, GAPDH and colormetric image of blot for molecular weight markers. Boxed bands are those used for publication.
B: Complete blots for p-p38 MAPK, total p38 MAPK, GAPDH and colormetric image of blot for molecular weight markers. Boxed bands are those used for publication.
C: Complete blots for COX-2, GAPDH and colormetric image of blot for molecular weight markers. Boxed bands are those used for publication.

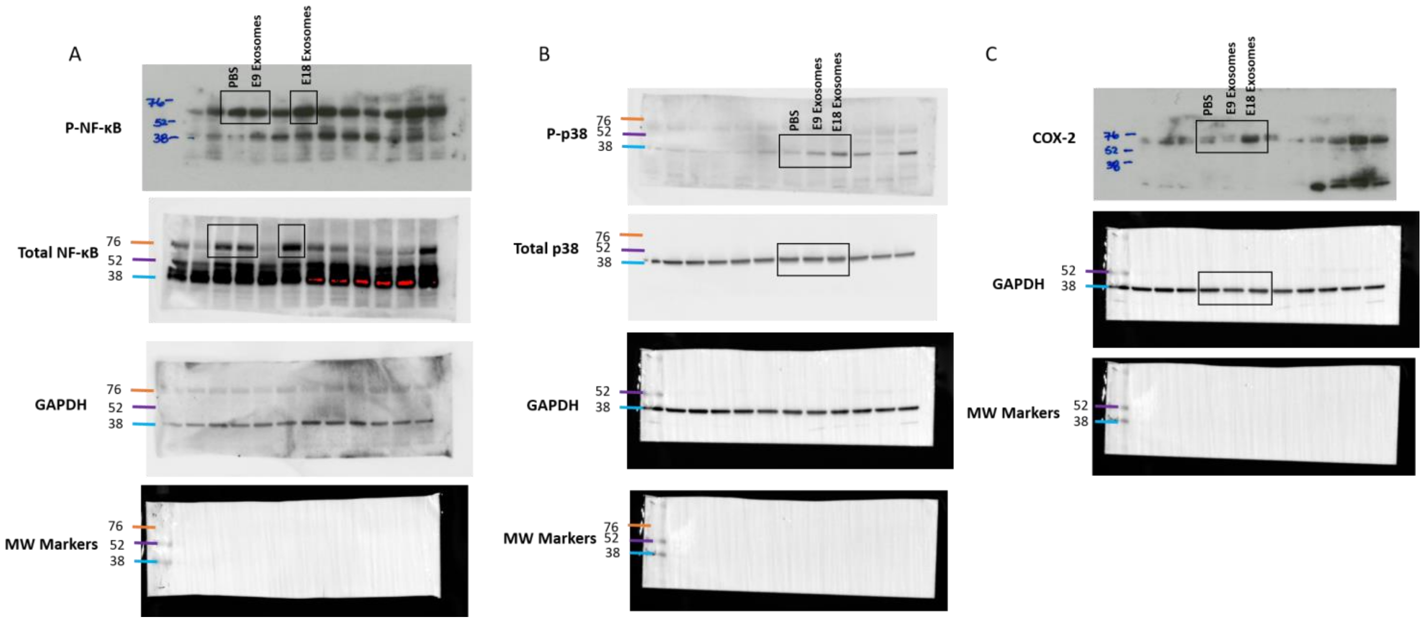

Supplemental Figure 6: **Full length blots of placenta tissues.**

A: Complete blots for p-NF-κB, total NF-κB, GAPDH and colormetric image of blot for molecular weight markers. Boxed bands are those used for publication.

B: Complete blots for p-p38 MAPK, total p38 MAPK, GAPDH and colormetric image of blot for molecular weight markers. Boxed bands are those used for publication.

C: Complete blots for COX-2, GAPDH and colormetric image of blot for molecular weight markers. Boxed bands are those used for publication.

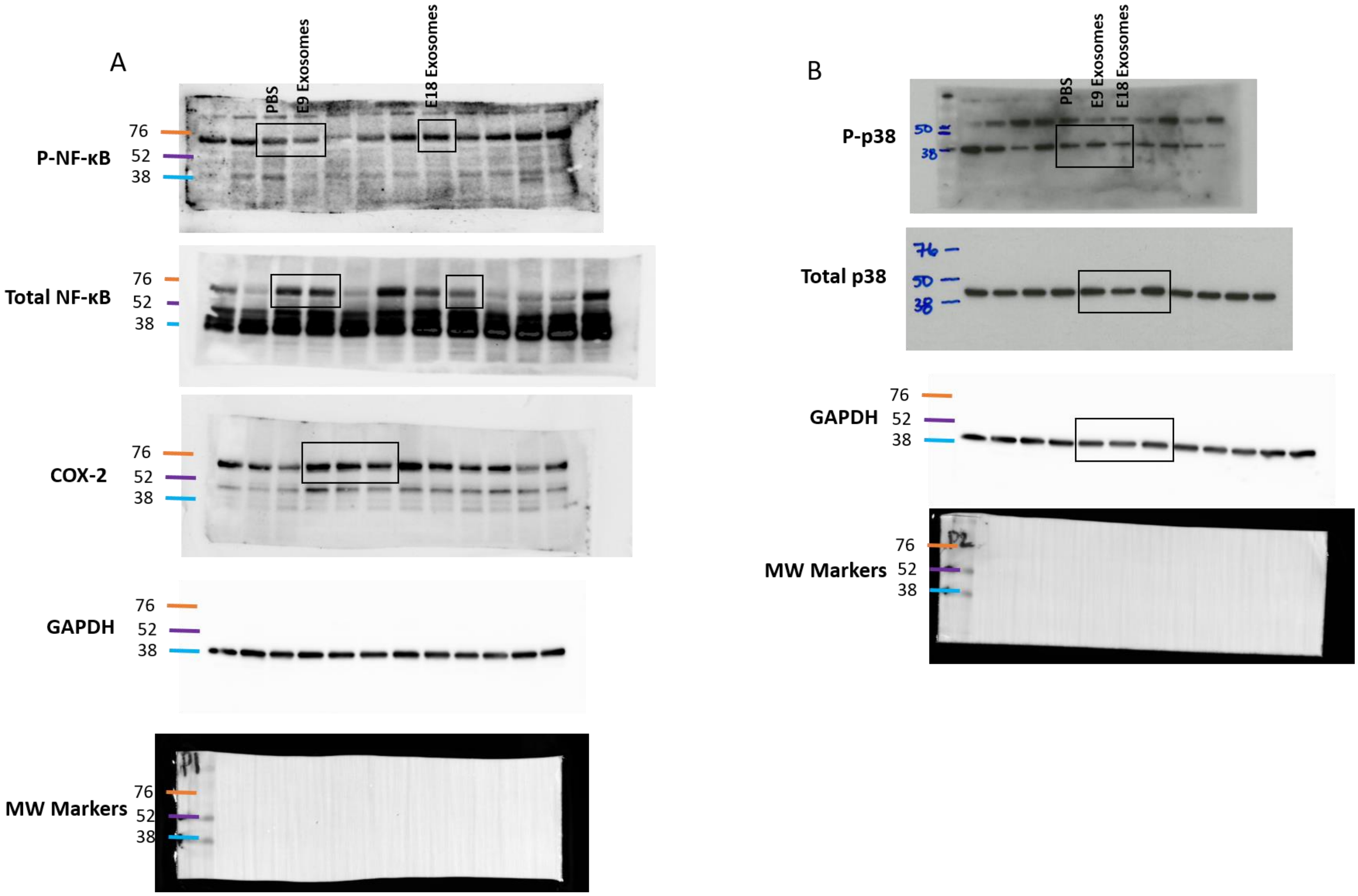

Supplement: Supplementary file 1 — Supplemental Tables and Figures [file 41598_2018_37002_MOESM1_ESM.pdf]
